# Mining Functional Modules in Heterogeneous Biological Networks Using Multiplex PageRank Approach

**DOI:** 10.3389/fpls.2016.00903

**Published:** 2016-06-22

**Authors:** Jun Li, Patrick X. Zhao

**Affiliations:** Bioinformatics Lab, Plant Biology Division, The Samuel Roberts Noble FoundationArdmore, OK, USA

**Keywords:** heterogeneous biological network, sub-network, functional module, multiplex PageRank, mPageRank, gene expression association network, protein-protein interaction network, *Arabidopsis thaliana*

## Abstract

Identification of functional modules/sub-networks in large-scale biological networks is one of the important research challenges in current bioinformatics and systems biology. Approaches have been developed to identify functional modules in single-class biological networks; however, methods for systematically and interactively mining multiple classes of heterogeneous biological networks are lacking. In this paper, we present a novel algorithm (called mPageRank) that utilizes the Multiplex PageRank approach to mine functional modules from two classes of biological networks. We demonstrate the capabilities of our approach by successfully mining functional biological modules through integrating expression-based gene-gene association networks and protein-protein interaction networks. We first compared the performance of our method with that of other methods using simulated data. We then applied our method to identify the cell division cycle related functional module and plant signaling defense-related functional module in the model plant *Arabidopsis thaliana*. Our results demonstrated that the mPageRank method is effective for mining sub-networks in both expression-based gene-gene association networks and protein-protein interaction networks, and has the potential to be adapted for the discovery of functional modules/sub-networks in other heterogeneous biological networks. The mPageRank executable program, source code, the datasets and results of the presented two case studies are publicly and freely available at http://plantgrn.noble.org/MPageRank/.

## Introduction

The advent of systems biology, which often integrates microarray- or RNA-Seq-based transcriptomics, proteomics, and metabolomics analyses, has made this an opportune time to determine how biological processes and complex phenotypes (also called traits) are regulated in living cells. In the post-genomics era, the development of high-throughput “omics” technologies has generated vast amounts of mRNA, protein, and metabolite profiles for many eukaryotic species, and much of this “big data” has been made publicly accessible through data repositories (Pruitt and Maglott, 2001; Parkinson et al., 2005; Barrett et al., 2007; Brandao et al., 2009). “Big data” has the potential to provide unprecedented insights into the various biological processes, including trait-regulation, leading to the discovery of vast amounts of novel biological information.

Bioinformatics and systems biology approaches, which include biological network analyses, have great potential to elucidate the fundamental mechanisms that govern dynamic cell organization and function. In the past decade, a number of experimental (Rual et al., 2005; Arabidopsis Interactome Mapping Consortium, 2011) and computational (Ma et al., 2007; Brandao et al., 2009; Stark et al., 2011; Li et al., 2013, 2014) approaches have been developed to generate and predict protein-protein interaction networks, transcriptional regulatory networks, gene-gene co-expression networks, and metabolic networks in humans, animals and plants. The current challenge, however, is how to effectively identify significant functional modules or sub-networks in these extensive global networks. Complex biological processes in livings cell are carried out through interactions between multiple functional modules at various levels (Barabasi and Oltvai, 2004; Cancer Genome Atlas Research Network, 2008), and members of the same functional module are often more densely connected than those across functional modules (Hartwell et al., 1999). Based on these observations, various clustering approaches, including hierarchical clustering, *k*-mean clustering, and Markov clustering, have been applied to identify function-specific modules in single-class biological networks (Eisen et al., 1998; Wu, 2008; Shih and Parthasarathy, 2012).

Due to the dynamic characteristics of living cells, networks generated from a single data source are usually limited, and can only reveal partial, static snapshots of the cell. Additionally, current technologies are often plagued by noise, leading to biases and low confidence in networks constructed by these technologies. To provide an accurate and comprehensive understanding of biological systems, the integration of different types of biological data has become an important and popular strategy. A number of approaches (Ideker et al., 2002; Chuang et al., 2007; Dittrich et al., 2008; Inoue et al., 2010) have been developed to facilitate the identification of context-dependent active functional modules/sub-networks by integrating protein-protein interaction (PPI) networks with gene expression data. On the basis of the topology structure of a PPI network, most of these methods first devise a function to score interacting edges or nodes in PPI networks while taking into account the gene expression data, and then employ optimization algorithms or graphical clustering algorithms to search the high-scoring modules/sub-networks (D'haeseleer et al., 2000; Enright et al., 2002; Langfelder and Horvath, 2008; Inoue et al., 2010). These methods have achieved some success in identifying biologically significant sub-networks, but have noticeable limitations. First, high confidence PPI networks are far from complete, especially in plants, and significant proportions of biologically significant genes/proteins may be overlooked in the PPI networks. For example, the PPI data included in the database of *Arabidopsis thaliana* protein interaction networks (Brandao et al., 2009) only include 201,699 interactions among 15,426 genes, while the genome of the organism encodes more than 20,000 genes. Second, cellular processes are the result of elegant coordination among gene regulation, signal transduction, and protein-protein interactions, etc. Some key proteins or genes that mediate or control these interactions among other cellular processes may be overlooked when utilizing a single network, even when the data are integrated with other types of biological data. Therefore, systematic and comprehensive mining of functional modules in heterogeneous biological networks, such as protein-protein interaction/protein-DNA interactions and gene-gene expression network, etc., is a better approach for understanding the complex mechanisms that govern the dynamic organization and function of living cells.

The Multiplex PageRank method (Halu et al., 2013), which is an extension of the widely used PageRank method (Langville and Meyer, 2006), leverages network transitivity to rank nodes in heterogeneous social networks. It simulates “bias random walking” on multiple networks to rank nodes in multiplex systems, in which the importance of a node in one network is affected by the importance that node gained in another network. The more important a node is in network A, the more important that node, and the nodes connected to it, in network B. The Multiplex PageRank method also considers the importance of the node's in-neighbors in the corresponding A/B network. The method has been shown to be highly accurate in community discovery in social networks (De Domenico et al., 2015). Recent studies (Girvan and Newman, 2002; Zhu et al., 2007; Vashisht et al., 2013) indicate significant similarities between biological networks and social networks in term of network properties, including the small-world property, power-law degree distribution, and network transitivity.

Here we present a novel method, named mPageRank, which adopts the Multiplex PageRank strategy to mine functional modules in heterogonous biological networks, including expression-based gene-gene association networks and protein-protein interaction networks. We demonstrated the effectiveness of our method by benchmarking against other existing methodologies, using both simulated data and experimental data. Compared with other methods and tools such as the jActiveModule method (Ideker et al., 2002), kwalks method (Faust et al., 2010), and DMSP method (Maraziotis et al., 2007), ours was the most accurate. We further demonstrated the effectiveness of our method by identifying cell division cycle related and plant signaling defense-related functional modules/sub-networks in *Arabidopsis thaliana*. These results suggest that the mPageRank is a promising approach for mining functional modules in heterogeneous biological networks.

## Materials and methods

### Analysis procedures

**Figure 1:**
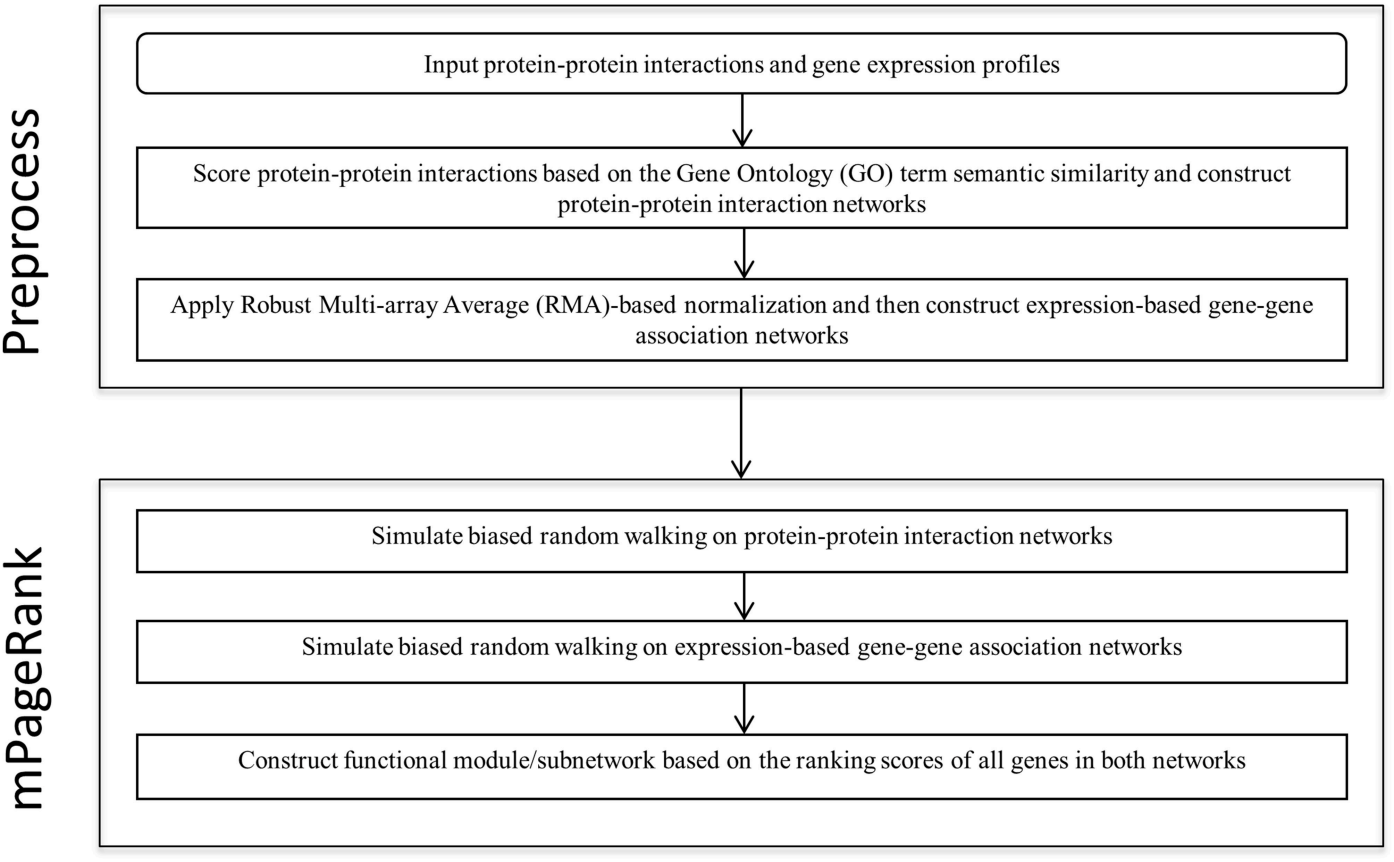
**The key steps in the functional module/sub-network identification analysis**.

We developed a novel Multiplex PageRank-based algorithm to extract biologically significant functional modules in heterogonous biological networks, including gene expression-based gene-gene association networks and protein-protein interaction networks. Starting from seed genes, we iteratively simulated biased random walks on a gene-gene association network and a protein-protein interaction network to prioritize those genes related to the seed genes. We then extract the sub-networks based on the rank scores of all the genes in both networks. The analysis flow is illustrated in the Figure 1.

### Construction of the expression-based gene-gene association networks

To construct gene expression-based gene-gene association networks for *Arabidopsis thaliana*, a total of 4162 microarray hybridization-based gene expression profiles were downloaded from the ArrayExpress data repository (Parkinson et al., 2005). The dataset was firstly normalized using the Robust Multi-array Averaging (RMA) method (Irizarry et al., 2003). The gene association networks, including 22,497 nodes (genes) and 2,106,763 edges (links between genes), were then reconstructed by our DeGNServer (Li et al., 2013), which is a powerful high performance web server developed for large-scale gene association network (GAN) construction and analysis. Reconstruction was performed using the Spearman-based Context Likelihood of Relatedness (CLR) with the z-score threshold set at 4.3.

### Scoring *Arabidopsis thaliana* protein-protein interactions based on the gene ontology (GO) term semantic similarity

The *Arabidopsis* protein-protein interaction dataset was downloaded from the AtPIN database-*Arabidopsis thaliana* protein interaction network (Brandao et al., 2009). The *Arabidopsis thaliana* gene ontology (GO) annotations were downloaded from https://www.arabidopsis.org/portals/genAnnotation/functional_annotation/go.jsp. The protein-protein binary interactions were scored with Resnik implemented in GoSemSim (Yu et al., 2010), which is an R package for weighing binary protein-protein interactions by measuring the semantic similarity among GO terms of gene products (GO-term based protein annotations).

### mPageRank method

Here, we define the expression-based gene-gene association network as network A and the protein-protein interaction network as network B.

We first constructed the transition matrix, *W*_*A*_, based on the gene-gene correlation values for network A, and the transition matrix, *W*_*B*_, based on the GO term semantic similarity scores for the proteins in the network. The transition probability from gene *i* to gene *j* was calculated using the following equation (Equation 1),

(1) $$\begin{array}{l} w\left(i,j\right)\;=\;\frac{c\left(i,j\right)}{\overset{n}{\underset{k=1}{\sum }}c\left(i,k\right)} \end{array}$$

where *c(i, j)* is the correlation value or similarity score between gene *i* and *j*; if there is no interaction between gene *i* and *j*, then *c(i, j)* is zero. *n* is the number of genes that interact with the gene *i*.

On network A, we performed multiple iterations of biased random walks to rank genes using the following equation (Equation 2),

(2) $$\begin{array}{l} x_{i}^{\left(n\right)}\;=\;\alpha_{A}\underset{j}{\sum }W_{A}\left(i,j\right)\;\times \;x_{j}^{\left(n-1\right)}\;+\;\left(1-\alpha_{A}\right)\;\times \;p\left(i\right) \end{array}$$

where *p*(*i*) is set to 1/*k* for each seed gene, with *k* indicating the number of seed genes, and where $x_{i}^{0}$ is set to 1/*m*, with *m* indicating the total number of genes in the network. Here, known genes in a biological pathway or a functional module may be used as the seed genes. *p*(*i*) specifies preference for node *i*. α_*A*_ denotes the probability of “returning” to one of the seed genes.

The error tolerance was defined as the Euclidean distance between the current iteration and the previous iteration: *Error tolerance* = *Distance* ($x_{i}^{\left(n\right)}$, $x_{i}^{\left(n-1\right)}$), where *n* is the number of iterations.

In our experiments, an error tolerance threshold at 1e-10 was empirically tested sufficient to reach the convergence. Therefore, the error tolerance threshold is set at 1e-10 by default in the software. However, a user defined error tolerance threshold is also allowed. The output rank score for each gene *i* was reflected as the node importance score, denoted $x_{i}^{\left(A\right)}$.

On network B, we performed the biased random walk iteration process to rank genes as applied to network A, using a similar equation (Equation 3, below). As with network A, iterations were performed until the error tolerance was less than 1e-10.

(3) $$\begin{array}{l} x_{i}^{\left(n\right)}=\alpha_{B}\underset{j}{\sum }x_{j}^{\left(A\right)}W_{B}\left(i,j\right)\times x_{j}^{\left(n-1\right)}+\left(1-\alpha_{B}\right)\times p\left(i\right) \end{array}$$

As in Equation 2, *p*(*i*) was set to 1/*k* for each seed gene, with *k* indicating the number of seed genes, and where $x_{i}^{0}$ is set to 1/*m*, with *m* indicating the total number of genes in the network. α_*A*_ and α_*B*_ were set to the value of 0.85 based on an empirical value (Halu et al., 2013). The output rank score for each gene *i* was reflected as the node importance score, denoted $x_{i}^{\left(AB\right)}$.

### Construction of functional module and *p*-value estimation

Based on a user-specified output number of top-ranking nodes, *n*, our algorithm first grouped the top ranked nodes (in both networks) associated with the seed genes by alternative random walks into a functional module. Then, the interactions with nodes included in the functional module were added to the module as edges to denote the interactions in the gene expression-based association networks or protein-protein interaction networks. Finally, to evaluate the significance of the functional module, one million of potential modules with same number of nodes were randomly sampled, and a one-sample *Z*-test was then applied to calculate the *Z*-score using the following equation (Equation 4):

(4) $$\begin{array}{l} Z-score\;=\;\left(\bar{x}-u\right)∕\left(\frac{\sigma }{\sqrt{n}}\right) \end{array}$$

The *Z*-score of the identified functional module was converted to a *p*-value for downstream analyses.

### Gene set enrichment analysis (GSEA)

Gene set enrichment analysis (GSEA) was performed using the online tool agriGo (Du et al., 2010) (http://bioinfo.cau.edu.cn/agriGO/). The significance of the GO term enrichment was determined using a Fisher's exact test with the entire *Arabidopsis thaliana* genome as the background reference. A *Yekutieli* correction was used to control for false positives.

## Results and discussion

### Performance benchmark analysis using simulation data

**Figure 2:**
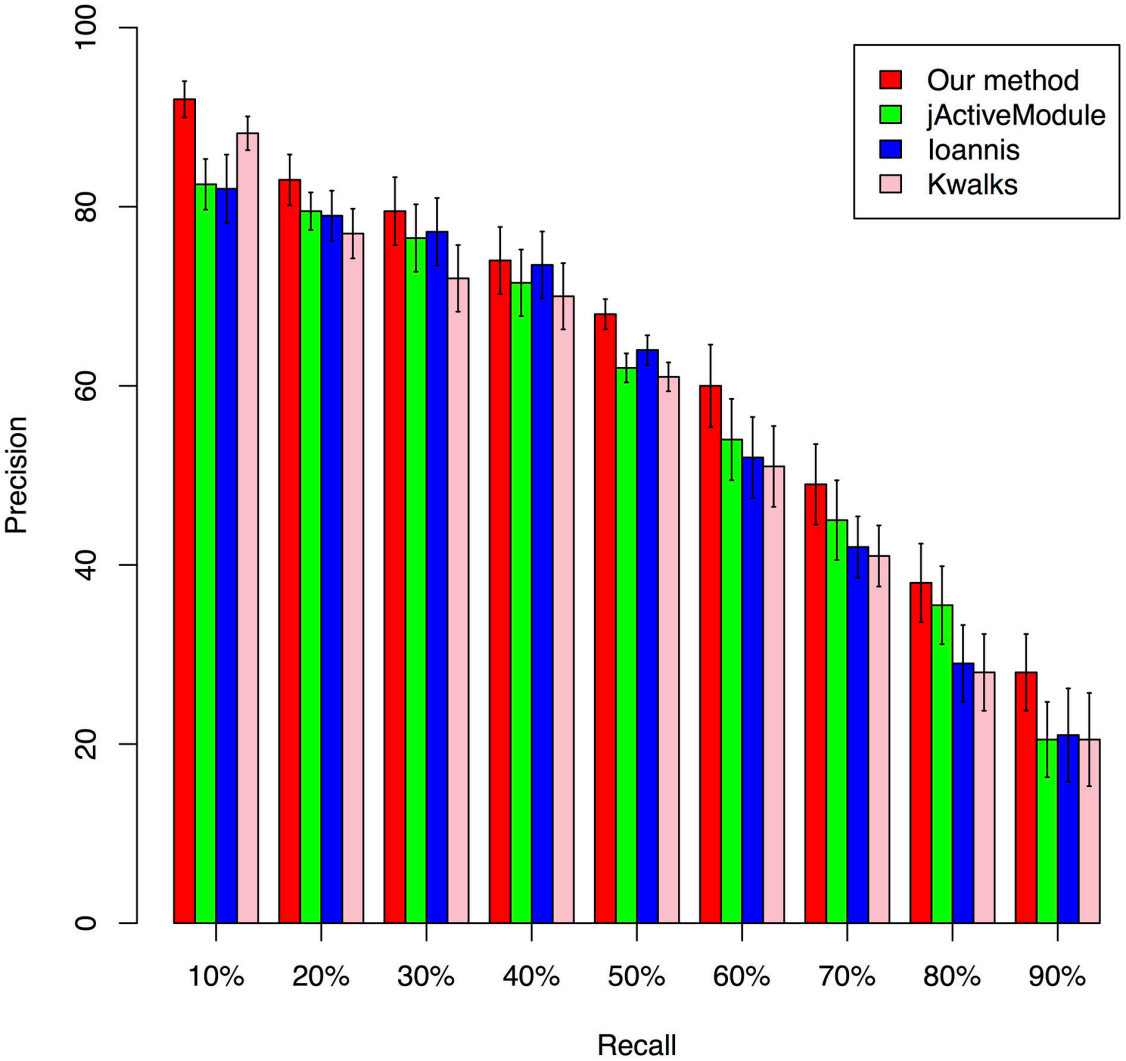
**Comparison of the precision of four different methods under different recall rates, ranging from 10 to 90%**.

We benchmarked the performance of our Multiplex PageRank-based method by comparing its performance against that of jActiveModule (Ideker et al., 2002), kwalks (Faust et al., 2010), and the method originally described by Ioannis et al. (Cancer Genome Atlas Research Network, 2008). The jActiveModule is a tool for module extraction from protein-protein interaction network through combining with expression profiles. In jActiveModule, the similarity for each interaction is measured with the expression value. The other two methods were developed based on the similar idea, but with different graph search strategies. These methods may have their advantages to identify those modules with high consistence between the expression profiles and PPI interactions network. However, such type of methods does not utilize the topological relationships inferred from expression profiles. In contrast, our mPageRank method effectively utilizes the topological relationship information via converting the expression profiles into gene-gene association networks, then walking on the two graphs (PPI networks and gene-gene association networks) to identify functional modules. Therefore, besides those interactions are highly consistent between two networks could be included, those interactions that are highly confident in a single network could also be included in the extracted subnetworks or functional modules. To generate simulation data for performance benchmark analysis, a yeast cell cycle pathway related module that included 113 genes and 369 experimentally validated interactions were downloaded from KEGG pathway database (Kanehisa et al., 2014) and used as the ground truth module for our benchmark performance analysis. We also extracted yeast cell cycle specific protein-protein interaction networks from the BioGRID database (Stark et al., 2011) Combining the two datasets, we created a network with 575 total genes and 803 total interactions, 685 of which were identified from protein-protein interactions. Due to the lack of experimentally validated expression datasets that could be applied to accurately extract all known interactions from this network, we utilized the widely used software, SynTren Van Den Bulcke et al., 2006, to generate a series of simulated expression datasets for performance benchmark analyses. We define as true positive (TP) a non-seed node that is present in both the reference pathway and in the inferred module, while a false positive (FP) was defined as a non-seed node found in the inferred module but not in the reference pathway. We defined a false negative (FN) as a non-seed node present in the reference pathway but not in the inferred module, while a true negative (TN) was defined as a node absent from both the inferred module and reference pathway. Using the true/false positive and true/false negative rates, the prediction accuracy of each method was evaluated by plotting Precision-Recall (PR) curves, where the Precision was calculated as *TP/(TP* + *FP)* and the Recall was calculated as *TP/(TP* + *FN)*. A series of functional modules were chosen with the variable sizes of the top-ranking nodes. The Precision and Recall value were calculated based on the confidence values of these series of functional modules. The average F-scores were calculated to estimate the accuracy (Supplemental Table 1). As illustrated in Figure 2, our method achieved the best precision under different recall rates, followed by the jActiveModule (Ideker et al., 2002) method, the Ioannis et al. method, and the kwalks method (Faust et al., 2010).

### Identification of a cell cycle core functional module

To further demonstrate the performance of our proposed method, we applied our method to a group of *Arabidopsis thaliana* specific datasets including gene expression-based association networks and protein-protein interaction networks to identify a cell cycle core functional module. The protein-protein interaction network was the same one as described in the previous section. An *Arabidopsis thaliana* gene expression-based association network comprised of 15,285 genes and 2,587,457 interactions was downloaded from http://plantgrn.noble.org/GPLEXUS/Result.jsp?sessionid=Arabidopsis.

Applying our method, we successfully baited a cell division cycle related core functional module that consisted of 70 genes and 403 interactions using the *KRP2* and *CDC2* genes as seed genes. The functions of the proteins encoded by these 70 genes, most of which are cell division cycle related (see the list in Supplemental Table 2). Among these 70 genes, 21 genes were identified as CDK genes or core cell cycle genes. Three E2F transcription factors were also included in the module. Other genes such as *WEE1, TON1, PAS2, AUR2*, and *SIM*, which have been validated to encode proteins with cell division cycle related functions (Bach et al., 2008; Gutierrez, 2009; Cook et al., 2013), were also included in the module.

**Figure 3:**
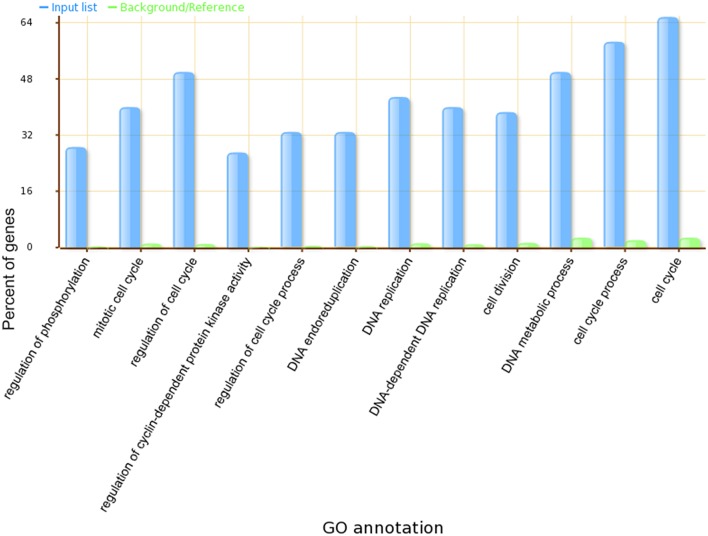
**The top 12 significantly enriched GO categories in our identified ***Arabidopsis thaliana*** cell cycle core functional module**.

A Gene Ontology Set Enrichment Analysis on the module (Supplemental Table 3) further demonstrated that the module we produced was composed of genes and proteins involved in the cell division cycle. Among the 70 genes in our module, 46 genes were annotated with cell cycle related gene ontology categories (under GO term: GO: 0007049) and an additional 35 genes were annotated with cell cycle regulation-related gene ontology categories (under GO term: GO:0051726). A comparison of the top 12 significantly enriched gene ontology categories in our module compared to the whole-genome GO categories is shown in Figure 3.

**Figure 4:**
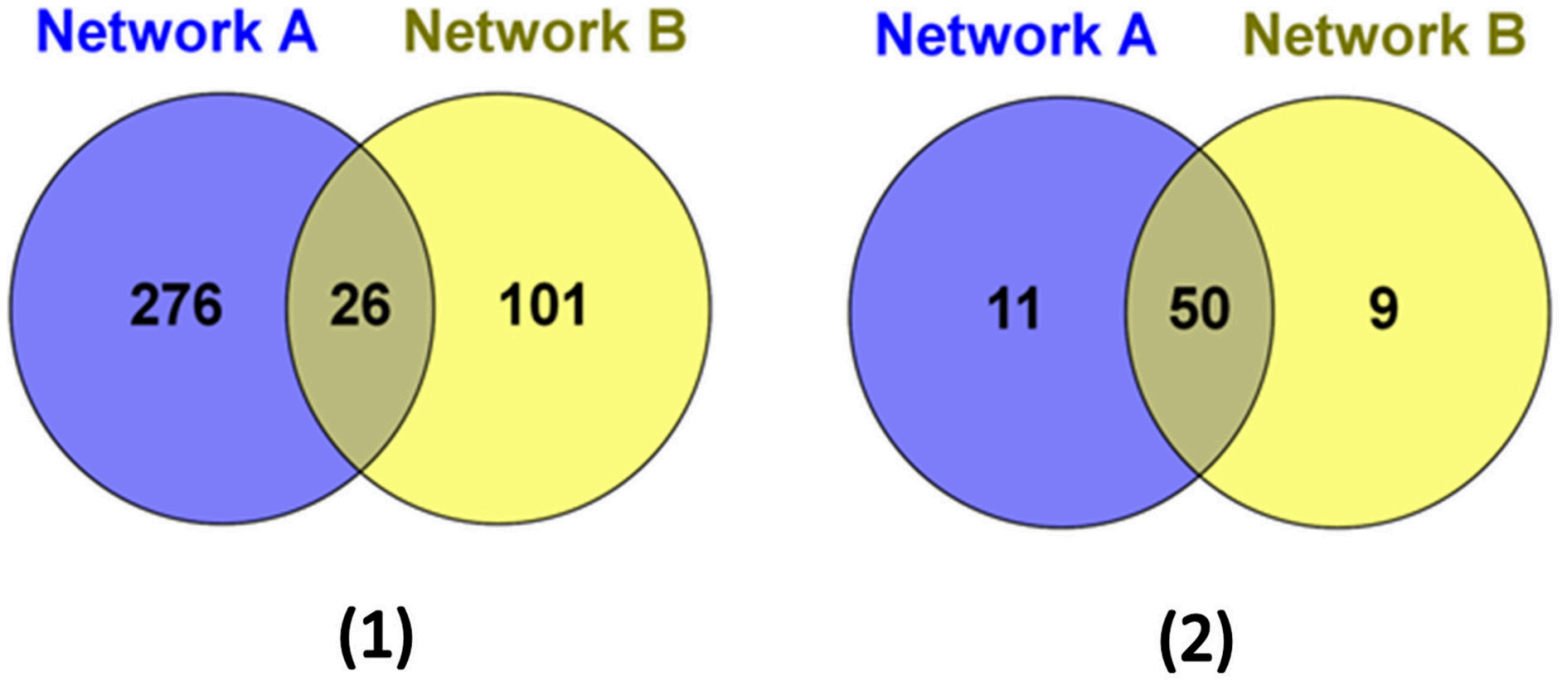
**The genes and interactions that were identified from the Network A: expression-based gene-gene association network and the Network B: protein-protein interaction network. (1)** The Venn Diagram shows the numbers of unique and shared genes in network A and B. **(2)** The Venn Diagram shows the numbers of unique and shared interactions in network A and B.

**Figure 5:**
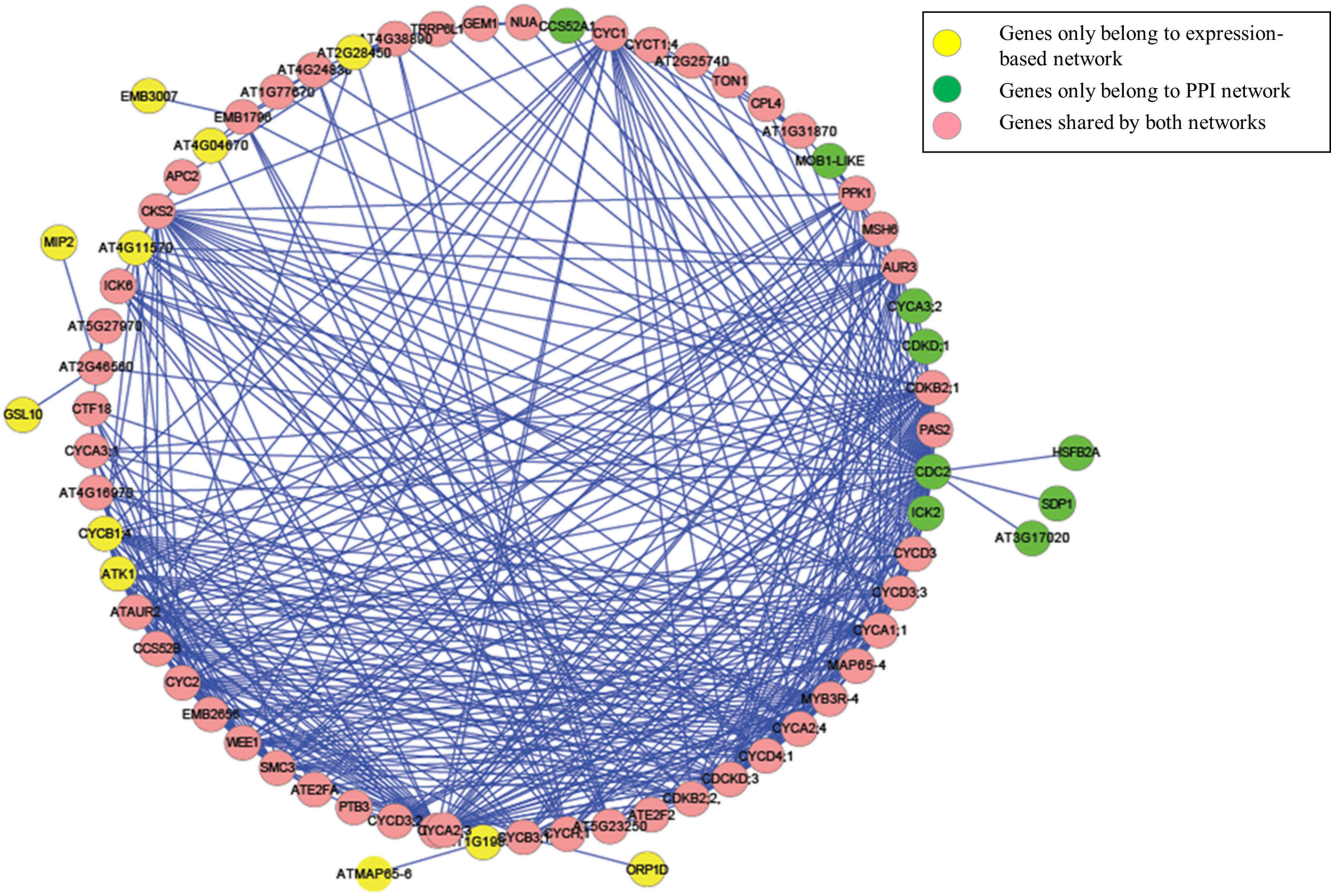
**The genes and interactions included in our identified ***Arabidopsis thaliana*** cell cycle core functional module**.

We then further examined the sub-network around the core genes in the module (Figures 4, 5). Among the 403 total interactions in the module, 276 were identified from the gene expression-based network and 101 from the protein-protein interactions network. Only 26 interactions were present in both networks (Figure 4A). This is not unexpected, as protein-protein interaction networks only reveal physical interactions, in contrast to expression-based gene-gene association networks, which not only reflect direct physical interaction, but also reveal potential cell regulatory relationships.

Although, the number of shared protein-protein interactions was minimal, most genes were present in both networks. As illustrated in Figure 4B, 61 genes were identified from the expression-based network and 59 were identified from the protein-protein network, while 50 genes were present in both networks. Importantly, we demonstrate that those genes existing in only one network would be missed using other methods (Ideker et al., 2002; Cancer Genome Atlas Research Network, 2008; Faust et al., 2010), which identify modules from a single network. These results suggest that mining the heterogonous datasets captured by multiple complementary technologies could provide greater insights in biology, leading to better reflection of the whole cellular activities.

We further examined the genes that were unique to each network. Among them, 11 (*CYCB1;4, EMB3007, GSL10, ATK1, AT4G04670, ORP1D, AT1G19835, ATMAP65-6, AT4G11570, AT2G28450*, and *MIP2*) could only be identified from the gene expression-based network, while 9 (*CYCA3;2, CCS52A1, AT3G17020, SDP1, CDC2, CDKD;1, ICK2, HSFB2A*, and *MOB1-LIKE*) could only be identified from protein-protein interaction network. Analyzing the functional descriptions of these genes indicates that all of these genes have been experimentally validated as cell cycle related genes. For example, *CYCB1;4* has been validated as the core cell cycle gene (Vandepoele et al., 2002), while *ATMAP65-6* encodes a protein that induces microtubules to form a mesh-like network (Mao et al., 2005). GSL10, a member of the glucan synthase-like (GSL) family, which is involved in synthesis of the cell-wall component callose at specialized locations, governs the entry of micropores into mitosis, and impaired GSL10 function leads to a perturbation in micropore division symmetry (Toller et al., 2008). Additionally, ATK1 plays a crucial role in spindle morphogenesis during meiosis in male *Arabidopsis* plants (Chen et al., 2002).

In the extracted module, among those identified interactions that could be experimentally validated, many of them only exist in one network. For example, the interaction between the cyclin-dependent protein kinase regulators *CYCA1;1* (*AT1G44110*) and *CYC1* (*AT4G37490*) was described in (De Almeida Engler et al., 2012), and the interaction between *CCS52B* and *CDKB2;2* (*AT1G20930*) was reported in Van Leene et al. (2010). Nevertheless, these interactions were only present in the gene expression-based association network but not in the protein-protein interaction network. On the other hand, the experimentally validated interactions between CDC2 and ATE2FA (Boruc et al., 2010), between CDC2 and TON1 (Van Leene et al., 2007), and between ICK6 and CKS2 (Van Leene et al., 2010) were only present in the protein-protein interaction network. Therefore, by performing biased random walks on both networks, we successfully identified an integrated cell division cycle functional module.

### Identification of an immune-related functional module involved in plant defense signaling

To further demonstrate the performance of our proposed method, we next applied our method to identify genes and sub-networks involved in the plant defense signaling. Plants have evolved highly sophisticated immune systems, which are critically important for plant survival in the face of life-threatening pathogens, pests, and harsh environmental challenges. Understanding the plant defense signaling pathway will help biologists to better understand the biotic and abiotic stress response mechanisms in plants. Using biased random walks with four plant seed genes (*FLS2, MPK4, WRKY40*, and *WRKY33*), we constructed a highly confident (*p* = 0.008) functional core module related to the MAMP signaling pathway (MAMP), the primary means by which plants detect and respond to pathogens.

Functional annotation of the genes present in this pathway revealed a number of biological features related to MAMP signaling pathway (see Supplemental Table 4 for a list of the functions of all genes in the module), providing strong evidence that the module is highly related to the plant defense signal transduction pathway. The major gene products included mitogen-activated protein (MAP) kinases, WRKY transcription factors, and vesicle-trafficking proteins, all of which have been reportedly involved in the plant defense signaling functions. Indeed, several MAP kinases involved in the plant defense response have been experimentally validated (Ligterink et al., 1997; Nuhse et al., 2000; Lee et al., 2001), and the WRKY transcription factors are involved in several immune responses in plants, including microbe-associated molecular pattern-*triggered* (MAMP-triggered) immunity, pathogen-associated molecular pattern-triggered (PAMP-triggered) immunity, effector-triggered immunity (ETI), and systemic acquired resistance (Eulgem et al., 2000; Xu et al., 2006; Encinas-Villarejo et al., 2009; Pandey and Somssich, 2009). We also identified BIK1, a positive regulator of plant immunity, and in our module, BIK1 functioned as a negative regulator of plant hormone brassinosteroid (BR)-mediated growth through association with the BR receptor BRI1. This dual association may contribute to the inverse functions of BIK1 previously reported in plant immunity and development (Lin et al., 2013).

**Figure 6:**
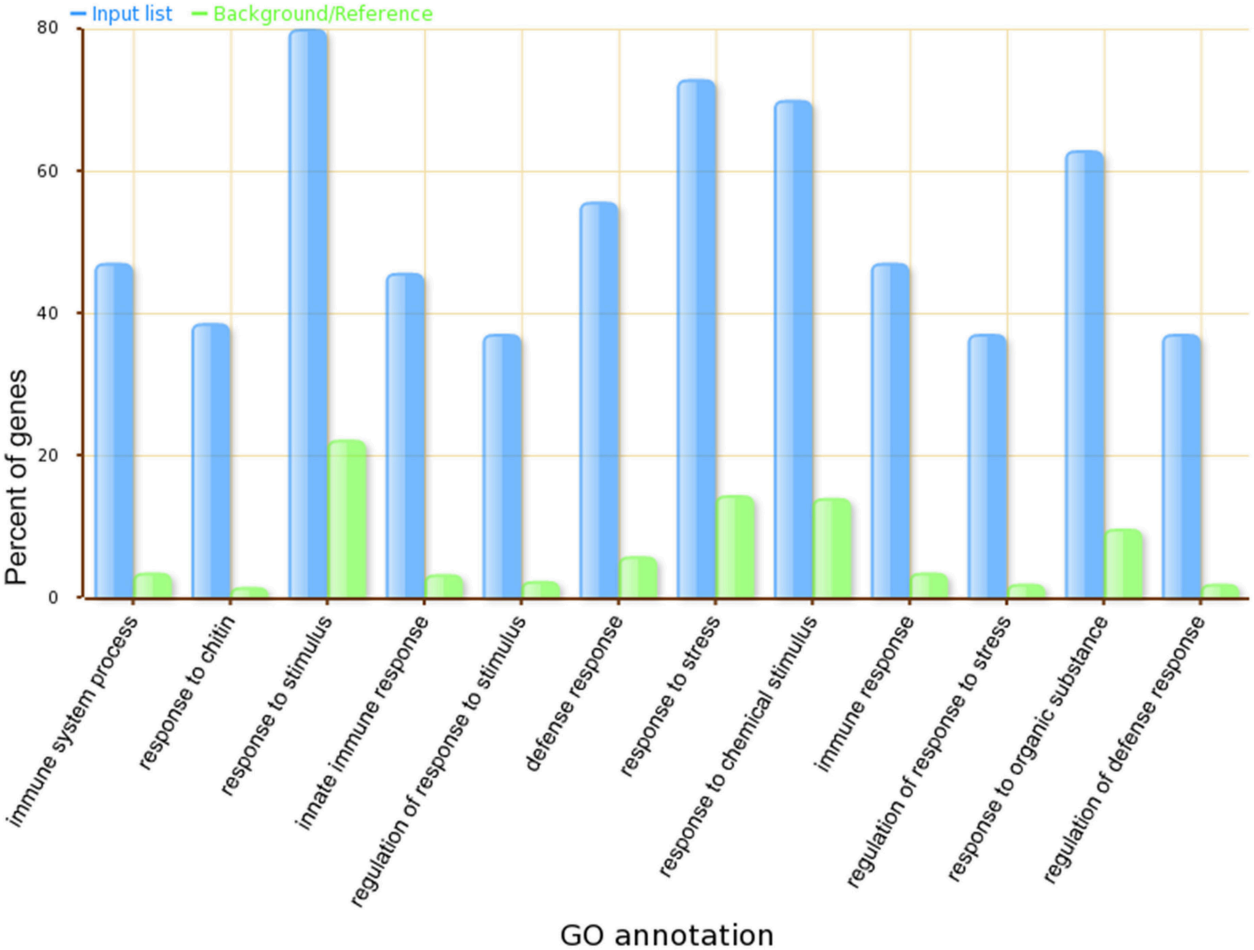
**The top 12 significantly enriched GO categories in our ***Arabidopsis thaliana*** defense response related functional module**.

GSEA (Supplemental Table 5) further validated that core genes in our module are highly related to plant defense signaling, as the GO terms defense response to bacteria, signaling transduction, cellular response to salicylic acid stimulus, and regulation of immune response were overrepresented. Almost 80 percent of genes in the module have the GO term referring to response to stimulus. A comparison of the top 12 significantly enriched gene ontology categories in our module compared to the whole-genome GO categories is shown in Figure 6.

**Figure 7:**
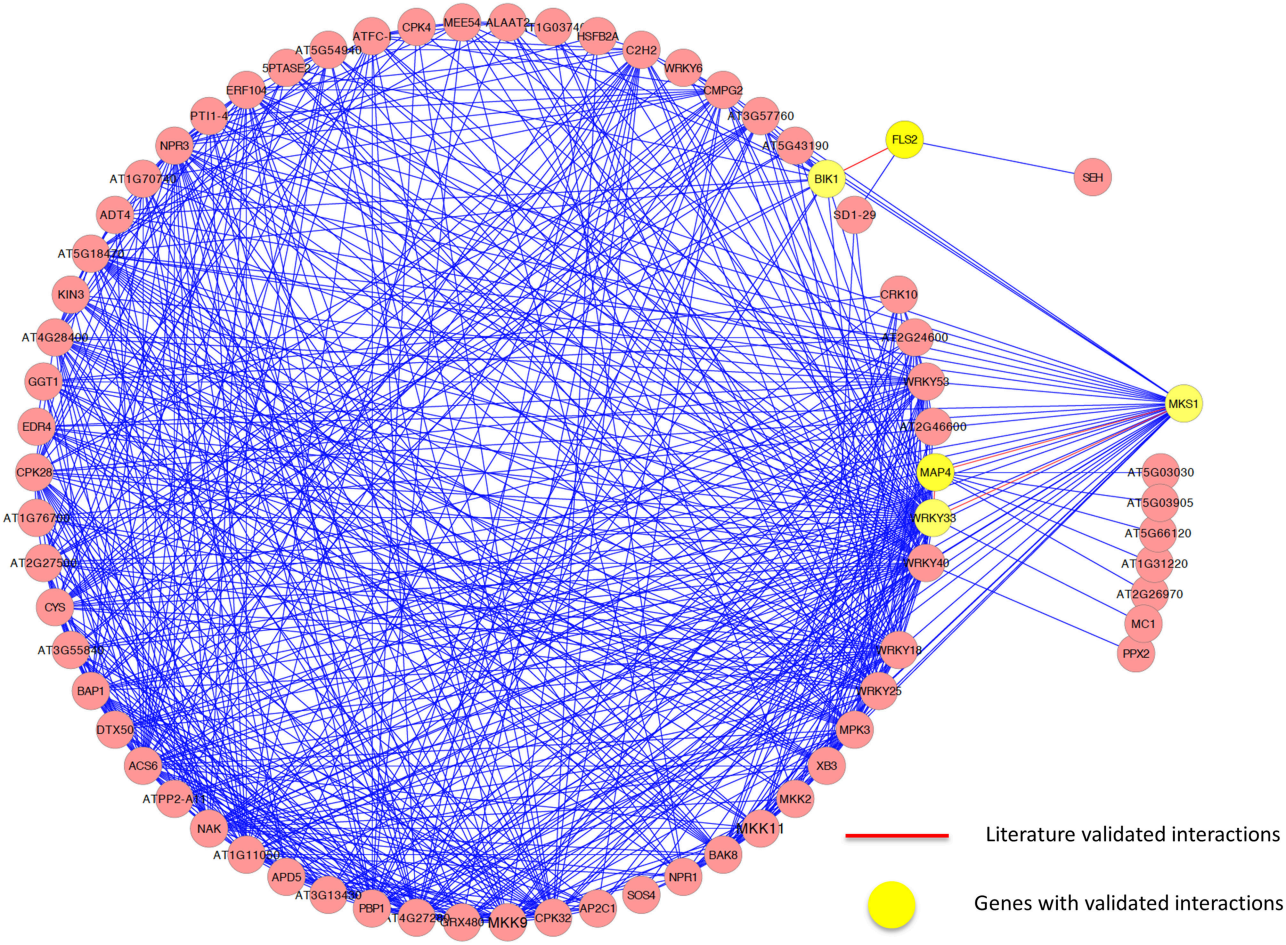
**The genes and interactions included in the identified functional core module related to defense response in ***Arabidopsis thaliana*****.

We further analyzed the sub-network around the core genes in the module and found several interactions that have been experimentally validated. Again, it demonstrates the effectiveness of our method. For example, it has been demonstrated that *in vivo*, FLS2 and BIK1 form a complex in a specific ligand-dependent manner, and that this interaction plays a functional role PRR-dependent signaling, which initiated innate immunity (Chinchilla et al., 2007). Qiu et al. (2008) demonstrated that WRKY33 can bind in a complex with MAP kinase 4 (MPK4) and MKS1, playing a role in the immune response to *Pseudomonas syringae*. While the interaction between WRKY33 and MPK4 could be revealed in the PPI network, the interaction between WRKY33 and MKS1 was identified in the expression-based network only. Using our method to integrate the two networks, both of these interactions were successfully identified. However, only the interaction between WRKY33 and MPK4 could be included by other three methods because they only considered those interactions in the PPI network. As with the cell division cycle functional module, some interactions were only identified in the expression-based gene-gene association network or the protein-protein interaction network, while some were identified in both networks. Figure 7 illustrates the core interactions of the functional core module, with experimentally validated interactions highlighted in red. Again, only part of those interactions that existed in a single network could be identified by other three methods due to their inability to combine the topological relationship information in both networks. Taken them together, we demonstrated that our mPageRank algorithm was effective in combining graph topological relationship information in two heterogonous biological networks for functional module discovery.

## Conclusions

We present here a novel mPageRank approach for mining functional modules in heterogeneous biological networks. Beginning with several cell division cycle related or immune-related seed genes from the model plant, *Arabidopsis thaliana*, our approach successfully ranked and retrieved genes involved in cell division cycle related functions and plant defense signaling related functions. These genes formed the basis for core functional modules created from global gene co-expression association networks and protein-protein interaction networks. Our benchmarking analyses using simulated data and case study analyses additionally demonstrated that our proposed mPageRank method is effective and is a promising approach for mining functional modules in heterogonous biological networks.

## Availability

The program namely mPageRank, datasets and results of the presented two case studies are publicly and freely available at http://plantgrn.noble.org/MPageRank/.

## Author contributions

JL implemented the mPageRank algorithm, carried out case study analyses and wrote the manuscript. PZ conceived and directed the study, performed bioinformatics analyses and wrote the manuscript.

## Funding

This work was supported by the National Science Foundation [grants DBI: 0960897 and DBI: 1458597 to PZ] and the Samuel Roberts Noble Foundation.

### Conflict of interest statement

The authors declare that the research was conducted in the absence of any commercial or financial relationships that could be construed as a potential conflict of interest.
